# Cdc45-MCM-GINS, a new power player for DNA replication

**DOI:** 10.1186/1747-1028-1-18

**Published:** 2006-08-24

**Authors:** Tomás Aparicio, Arkaitz Ibarra, Juan Méndez

**Affiliations:** 1 DNA replication Group, Molecular Oncology Programme, Spanish National Cancer Center (CNIO), Melchor Fernandez Almagro 3, E-28029 Madrid, Spain

## Abstract

The identity of the DNA helicase(s) involved in eukaryotic DNA replication is still a matter of debate, but the mini-chromosome maintenance (MCM) proteins are the chief candidate. Six conserved MCM proteins, Mcm2–7, are essential for the initiation and elongation stages of DNA replication, contain ATP binding pockets and can form a hexameric structure resembling that of known prokaryotic and viral helicases. However, biochemical proof of their presumed function has remained elusive. Several recent reports confirm that the MCM complex is part of the cellular machine responsible for the unwinding of DNA during S phase. In one of these reports, the helicase activity of Mcm2–7 is finally revealed, when they are purified in association with two partners: initiation factor Cdc45 and a four-subunit complex called GINS. The Cdc45-MCM-GINS complex could constitute the core of a larger macromolecular structure that has been termed the "replisome progression complex".

## Background

The biochemistry of DNA replication has been under intense scrutiny since the discovery of the double helix structure. The pioneering work of Arthur Kornberg led to the purification of the key elements involved in the duplication of bacterial genomes: "initiator" proteins that recognize and activate the origin of replication and a large DNA polymerase holoenzyme that synthesizes new polynucleotide chains. Subsequent research about DNA replication in other systems revealed a striking functional conservation of these elements between prokarya and eukarya [1]. In the case of mammalian cells, the biochemical studies largely relied on *in vitro *assays that reproduced the duplication of viral DNAs such as SV40 in cell extracts. SV40 DNA replication depends on host cell factors, with one important exception: it encodes its own initiator protein, the large T antigen, which also serves as DNA helicase [2]. For this reason, these studies failed to identify the replicative helicase required for the duplication of the cellular DNA.

In fact, the human genome encodes multiple DNA helicases that participate in different aspects of DNA metabolism. Mutations in some of them (e.g. WRN, BLM) result in human syndromes associated to increased genomic instability [3], but they are not essential for DNA replication. Replicative helicases are expected to start to unwind DNA from the origins of replication, discrete points at which "pre-replication complexes" (pre-RC) are assembled during G1. Pre-RC formation is mediated by the origin recognition complex (ORC), that in combination with Cdc6 and Cdt1 proteins, recruits the mini-chromosome maintenance (MCM) proteins to the DNA [2,4-6].

MCM genes were originally identified in a screening for mutants that affected plasmid maintenance in yeast [7]. The MCM protein complex was identified independently as part of the "licensing factor" needed to replicate chromatin DNA in Xenopus egg extracts [8]. MCM proteins are loaded onto the origins during G1 and appear to move with the forks after the G1-S transition [9]. They dissociate from the chromatin as the DNA is duplicated and their re-association during G2 is carefully prevented [10]. Also from the biochemical point of view, the MCM complex appears to be a good fit for the helicase role: it is composed by six ATPase subunits (Mcm2–7) assembled in a hexameric complex, a common structural arrangement of replicative helicases.

However, the helicase hypothesis is challenged by two intriguing facts. First, the majority of chromatin-associated MCM complexes are not present at the sites of DNA synthesis. Second, there is little evidence of DNA helicase activity associated with purified Mcm2–7 proteins. The first issue could be solved by arguing that only a small fraction of the MCM complexes are located at the forks, but are sufficient to carry out DNA unwinding. In this model, the "excess" MCM proteins would constitute a reserve to be used only under conditions of replication stress [11]. Another theory is that MCMs could operate away from the replication foci, somehow spooling ssDNA chains over to distant replisome complexes [12,13]. Regarding the lack of demonstrable helicase activity in purified Mcm2–7, it could be explained by the need of accessory factors. Two such auxiliary factors have now been identified: Cdc45 and GINS, a four-protein complex which possibly plays additional roles during DNA replication.

## Discussion

### The mystery of the precarious MCM helicase. A case of missing partners?

The experimental attempts to find helicase activity in MCM proteins from different organisms have yielded an array of disparate results. On one hand, three independent studies reported a relatively strong activity in the archaeal *Methanobacterium thermoautotrophicum *MCM, a single polypeptide that can multimerize to form hexamers and dodecamers. MtMCM could displace up to 200–500 nucleotides of DNA, translocating with 3'-5' polarity [14-16]. In contrast, biochemical analyses of yeast Mcm2–7 have failed to detect any helicase activity. Certain subunits (Mcm4, 6 and 7) can hydrolyze ATP as long as they are associated with one of the others, Mcm2, 3 or 5 [17]. This distinction between "catalytic" and "regulatory" MCM subunits is interesting because a Mcm4–6–7 subcomplex isolated from HeLa extracts showed a modest but reproducible oligonucleotide-displacement activity, whereas the full Mcm2–7 complex did not [18]. Finally, a timid strand-displacement activity has been observed in a complex formed by Xenopus Mcm2–7 and replication factor Cdc45 [19].

Many of these contrasting results could be reconciled by postulating that Mcm2–7 proteins are at the core of a macromolecular machine responsible for DNA unwinding during replication. *In vitro*, the unwinding efficiency would be reduced or even lost if the putative complex falls apart during purification, separating the MCM component from its necessary cofactors. In archaea, the "MCM core" on its own seems sufficiently strong to produce strand displacement; in Xenopus, Cdc45 could be one such cofactor; in other eukaryotic systems, additional proteins would be required. The identification of MCM-based DNA unwinding machines has become a hot topic in the field.

### New protein complexes at the sites of DNA unwinding

Several recent studies have confirmed that a fraction of the chromatin-bound MCM complexes are part of the replisome structure. Taking advantage of the existence of natural replication fork barriers (RFBs) at defined points of the yeast chromosomes, Calzada *et al *[20] engineered a yeast strain in which two such barriers are located next to ARS305 and ARS306, two very early origins of replication. In the presence of the fork barrier protein Fob1, replication forks emerging from the nearby origins are paused at the RFBs, and the components of the paused replisomes can be detected by chromatin immunoprecipitation (ChIP). Proteins that stabilize stalled forks such as Mrc1 and Tof1 [21] were detected, together with DNA polymerase ε, Mcm4, Cdc45 and Psf2 proteins [20]. Cdc45 is a known initiator protein [22] that seems to move with the replication forks [9]. Psf2 is part of GINS, a recently identified complex formed by proteins Sld5, Psf1, Psf2 and Psf3. GINS was discovered through genetic screenings aimed at the identification of novel replication factors [[23]; see below], and three of its components were identified independently with a functional proteomic approach [24].

In an interesting variation of the fork barrier scheme, Pacek *et al *[25] modified a circular DNA plasmid with biotin-streptavidin to generate a fork pausing site. During replication of this plasmid in a Xenopus egg extract, the replisome components are trapped at this site and can be identified by ChIP. Once again, replicative DNA polymerases were recovered, together with several MCM subunits, Cdc45 and GINS. An important result was obtained in the presence of aphidicolin, a DNA polymerase inhibitor: MCM, Cdc45 and GINS were still found at the pause site, whereas replicative polymerases were delocalized along the DNA. This result suggested a possible uncoupling between the DNA unwinding machinery, referred to as "unwindosome", and the actual replicative machinery [25]. The uncoupling of helicase and polymerase activities could trigger the cellular checkpoints in response to replicative stress [26].

### Capturing the "replisome progression complex"

A physical interaction between MCM, Cdc45 and GINS proteins was originally suggested by their co-immunoprecipitation in Xenopus egg extracts [27]. Two recent large-scale biochemical purifications carried out in yeast and Drosophila have confirmed this result and emphasized the importance of the MCM-Cdc45-GINS complex.

In the first study, a yeast strain expressing TAP-tagged Sld5 and Flag-tagged Mcm4 was created to facilitate the purification of protein complexes containing both MCM and GINS. The result was a large protein structure (>1400 KD) that assembles specifically during S phase and has been termed "replisome progression complex" or RPC [28]. The main components of RPC, identified by mass spectrometry, turned out to be Mcm2–7, Cdc45 and GINS, in association with Mrc1, Tof1, Csm3 (involved in the stabilization of stalled forks), Ctf4 (required for sister chromatid cohesion), Spt16 and Pob3 (components of the histone chaperone FACT). The majority of these proteins formed a stable complex even in the presence of high salt concentrations. In addition, other replication factors like Top1 (topoisomerase I) and Mcm10 (a putative primase; [29]) associated weakly with the RPC. GINS appears to be a central component of the RPC, serving as a molecular link between Cdc45 and the MCM proteins. When Psf2 was eliminated from the yeast extracts, RPC failed to form although MCM could still associate with Mcm10 or subunits of FACT [28].

It is remarkable that no DNA polymerases or accessory factors like PCNA were part of the RPC. Therefore, the RPC consists of the "unwindosome" described in Xenopus (see above) plus a group of additional factors that might deal with nucleosome structure coupled to replication (FACT histone chaperone) and possibly facilitate the establishment of sister chromatid cohesion after passage of the fork (Ctf4).

### CMG: a purified Cdc45-MCM-GINS complex with helicase activity

In the second study, Moyer *et al *[30] carried out a purification of Cdc45 from Drosophila embryo extracts, consisting of several chromatography steps followed by a Cdc45 immunoprecipitation. The result was an eleven-member complex consisting precisely of Cdc45, the six MCM and the four GINS components. The complex was hence called CMG. An alternative purification was performed with flies expressing a Flag-tagged Mcm6: the Flag-affinity purification step followed by a Cdc45 immunoprecipitation yielded the pure CMG complex again. This study has gone one step further, by providing biochemical proof that CMG contains helicase activity *in vitro*. Purified CMG was able to displace a 40 nt oligonucleotide hybridized to a ssDNA plasmid, in an ATP-dependent manner. It should be reminded that until now, no helicase activity associated with the entire Mcm2–7 complex had been reported. As expected from their presumed role as part of the replicative helicase, Cdc45 and GINS are essential for cell cycle progression in Drosophila [30].

### GINS functions: the unwindosome and beyond

**Figure 1:**
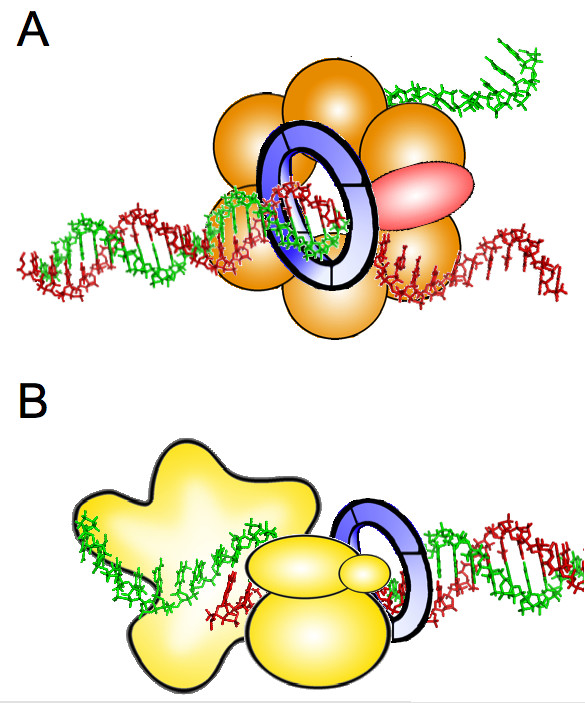
**Possible roles of GINS during DNA replication**. **A**. Structural component of the CMG complex that contains DNA helicase activity. Cdc45 protein is represented in red, Mcm2–7 proteins in orange, and GINS as a blue ring. **B**. DNA polymerase ε auxiliary factor. The different subunits of Pol ε are depicted in yellow. The relative position of the different components in each protein complex is speculative. See text for details.

Both Cdc45 and GINS appear to enhance the activity of the Mcm2–7 complex: they could do so by providing structural elements that improve its interaction with the DNA, translocation speed, or base pair separation activity. While no structural information is available about Cdc45, electron microscopy analyses suggest that GINS is a ring-shaped molecule [27]. GINS could overlap in the CMG complex with the ring-shaped MCM hexamer, and the resultant "fellowship of rings" could secure the interaction of the helicase with one or both strands of the DNA molecule [[30]; see Figure 1A].

GINS structure is also reminiscent of that of PCNA, the known processivity factor for replicative DNA polymerases. It should be noted that the lineage of genetic screenings that led to the discovery of GINS started with DNA polymerase ε: Dpb11 was first identified as a multicopy suppressor of Pol ε mutations [31], and several SLD genes were identified because they displayed synthetic lethality with a Dpb11 mutation [32]. Finally, Sld5 allowed the identification of Psf1, 2 and 3 (partners of Sld-five) by genetic and biochemical methods [23]. Therefore it is tempting to speculate that GINS could be an accessory factor for DNA polymerase ε, possibly a "sliding clamp". Indeed, a recent report shows that in budding yeast, GINS forms a complex with DNA polymerase ε and enhances its activity *in vitro *[[33]; see Figure 1B].

The functions of GINS proposed in Figure 1 are not mutually exclusive. If both were to occur coordinately, GINS would provide a link between the processes of DNA unwinding and polymerization. Additional functions at the replication fork are possible: an archaeal GINS homolog can associate with the MCM helicase and the DNA primase, suggesting that it coordinates MCM progression at the leading strand with priming events at the lagging strand [34].

## Conclusion and open questions

Recent biochemical studies have led to the discovery of CMG, a complex formed by Cdc45, Mcm2–7 and GINS, in different organisms. CMG is present at the sites of DNA unwinding during replication and contains an intrinsic helicase activity. This finding alleviates some nagging issues about MCM function that have lingered in the DNA replication field for a long time.

However, the case of the replicative helicase should not be closed yet. The apparent impossibility to detect MCM proteins at DNA replication foci by immunofluorescence remains unexplained. Also, the processivity of CMG helicase was not significantly higher than that of Mcm4–6–7 subcomplex alone [30]. In addition, vertebrate organisms contain another member of the MCM family, Mcm8, which at least in Xenopus egg extracts appears to function as a helicase independently of Mcm2–7 [35]. The possible coordination of Mcm8 and CMG activities needs to be explored.

Finally, there is limited information about GINS and the hypothetical CMG complex in mammalian cells. Human GINS proteins form a stable tetramer that is essential for DNA replication and cell cycle progression (our unpublished observations). A Psf1 KO mouse results in early embryonic lethality due to defective cell proliferation [36]. It will be exciting to determine whether mutations or partial depletion of these important proteins may affect genomic stability. Conditional *knock-out *mouse models will ultimately help to determine the possible implication of any of them in carcinogenesis.

## Abbreviations

ChIP: Chromatin immuno-precipitation; CMG: CDC45-MCM-GINS complex; GINS: Sld5 (go), Psf1 (ichi), Psf2 (nii), Psf3 (san) complex; MCM: mini-chromosome maintenance; pre-RC: pre-replication complex; RPC: replisome progression complex.

## Competing interests

The author(s) declare that they have no competing interests.

## Authors' contributions

TA, AI and JM contributed to the preparation of this article with ideas and discussions. The text was written by JM. All authors read and approved the text.
